# Uprighting an Impacted Permanent Mandibular First Molar Associated with a Dentigerous Cyst and a Missing Second Mandibular Molar—A Case Report

**DOI:** 10.3390/dj7030063

**Published:** 2019-06-27

**Authors:** Konstantina Tsironi, Emmanouil Inglezos, Emmanouil Vardas, Anastasia Mitsea

**Affiliations:** 1 Posidonos 14, Imia square, Voula, 16673 Athens, Greece; 2 Clinic of Hospital Dentistry, Dental School, National and Kapodistrian University of Athens, Thivon 2 Goudi, 11527 Athens, Greece; 3 Department of Oral Diagnosis and Radiology, Dental School, National and Kapodistrian University of Athens, Thivon 2 Goudi, 11527 Athens, Greece

**Keywords:** first molar uprighting, impacted molar, dentigerous cyst, unerupted tooth, missing second mandibular molar

## Abstract

The purpose of this paper is to present a case of an impacted mandibular first molar associated with a dentigerous cyst and a missing mandibular second molar in an 11-year-old girl that was treated with combined surgical and orthodontic procedures. After clinical and radiographic evaluation, marsupialization of the cyst was decided, and a molar attachment was bonded on the buccal side of the impacted molar as a part of a full orthodontic treatment with fixed appliances. After 18 months of orthodontic traction, the molar was moved to a more advantageous position, and new bone apposition was observed on the site of the cystic lesion. Histological examination confirmed a dentigerous cyst. The molar was left to erupt spontaneously for 14 more months. A functional occlusion was finally achieved. An interdisciplinary approach proved to be an effective modality in treating a large dentigerous cyst associated with a deeply impacted first mandibular molar, presenting many advantages, such as new bone apposition and patient comfort.

## 1. Introduction

Tooth impaction can be defined as tooth retention due to an obstacle in the eruption path or—less commonly—due to an ectopic position of the tooth germ [1]. Tooth impaction is relatively common (prevalence of 17%) [2], third molars being the most commonly affected teeth, followed by maxillary canines, mandibular premolars, and mandibular canines. The prevalence rate of impaction for the mandibular second molar ranges from 0.06 to 0.3%, and for the mandibular first molar, the prevalence described is <0.01% [3]. Impaction of the first molar is a serious problem that needs to be addressed in order to achieve functional occlusion and facial harmony. In cases of non-treatment, it can cause a decrease in the vertical dimension of the lower face, malocclusion, extrusion of the antagonist, root resorption in the adjacent teeth, or formation of a dentigerous cyst [4,5].

Dentigerous cysts are odontogenic cysts that originate by separation of the follicle from around the crown of an unerupted tooth [6]. Generally, they are associated with the crowns of impacted or unerupted permanent teeth or, less frequently, with an odontoma, a developing tooth, or a deciduous tooth [7,8]. The incidence of dentigerous cysts in the general population has been estimated at 1.44 cysts for every 100 unerupted teeth [9], comprising the second most common (14 to 24%) of all odontogenic cysts [10,11]. Most often, dentigerous cysts present no clinical symptoms and are detected during routine radiographic examination. In some cases, gum swelling or sensitivity, tooth mobility, and displacement of adjacent teeth may be observed if the cyst reaches large dimensions (>2 cm in diameter) or if it gets infected [6,12]. Radiographically, dentigerous cysts are characterized by a symmetric, well-circumscribed radiolucent lesion, most often unilocular, surrounding the crown of an unerupted tooth [12,13]. Differential diagnosis from other cysts, such as radicular cysts and odontogenic keratocysts, or from tumors, such as ameloblastoma, calcifying epithelial odontogenic tumor, and odontogenic fibroma, is necessary through histopathologic evaluation [14]. Dentigerous cysts are generally treated surgically either by enucleation, marsupialization, or by decompression of the cyst via fenestration [15,16,17]. This case report describes a conservative surgical approach combined with orthodontic treatment of an impacted first mandibular molar associated with a dentigerous cyst in an adolescent.

## 2. Case Presentation

An 11-year old female came to the clinic after her parents complained of missing lower left teeth. No pain or previous discomfort was reported. The overall patient’s dental and physical health was good with non-specific general medical history and no contra-indication to dental treatment. A signed informed consent from the patient’s mother was obtained before the patient participated in the study.

Extraoral examination revealed a symmetric face with no deficit in the lower left part of the face. Intraoral examination revealed a Class II incisor relationship and a Class II molar relationship from the right side in a late mixed dentition. At the left side, the first mandibular molar was clinically absent, and the overlying mucosa was normal in color and texture. The adjacent deciduous second molar had a large amalgam restoration with no signs of secondary caries.

**Figure 1:**
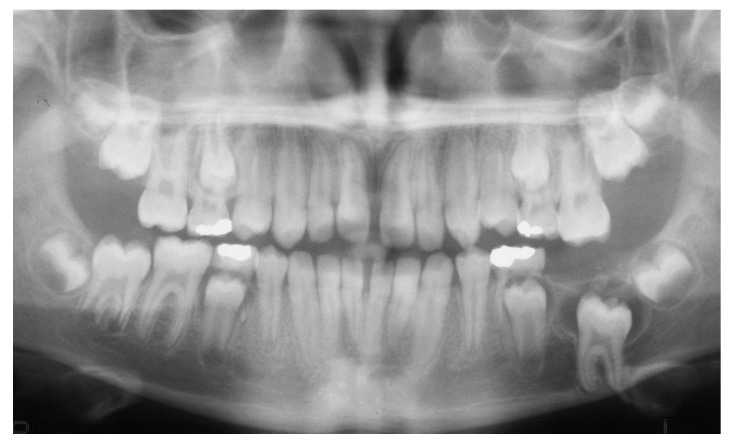
Pretreatment panoramic radiograph showing an impacted mandibular left molar associated with a rather large, well circumscribed, unilocular radiolucent lesion. The root is almost fully developed.

**Figure 2:**
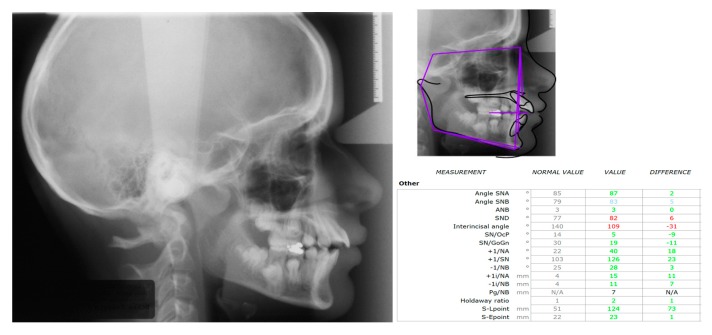
Pretreatment cephalometric radiograph showing a skeletal Class II profile.

The panoramic radiographic examination (PanRad) revealed the presence of six permanent molars in the upper jaw and five permanent molars in the lower jaw (Figure 1). From the size of the teeth, the stage of the root formation, the location of the teeth buds, and the angulation of the impacted molar, it was assumed that the impacted tooth was the first mandibular molar, and the adjacent tooth bud was the mandibular third molar. A well-circumscribed unilocular radiolucent lesion in the body of the mandible was noticed, associated with the crown of the vertically impacted mandibular left first molar. The roots of the impacted molar were completely developed with closed apexes. The cephalometric X-ray confirmed a skeletal Class II malocclusion (Figure 2). The clinical diagnosis was dentigerous cyst associated with the impacted molar.

**Figure 3:**
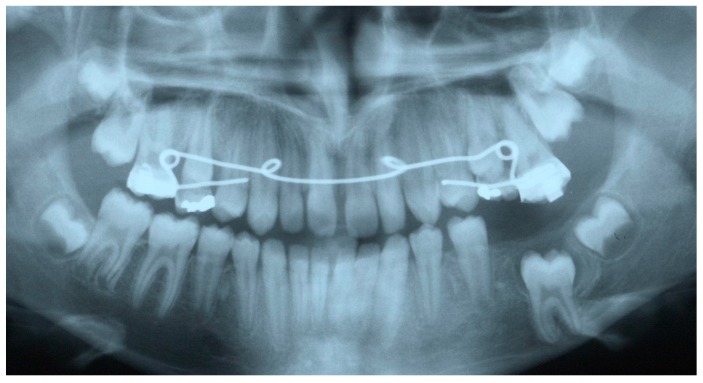
Panoramic radiograph after the extraction of the mandibular deciduous molars with the Quad-helix appliance.

The main objectives of the treatment plan were to eliminate the cystic lesion and establish a functional occlusion. The latter should include expansion of the upper arch, leveling and alignment of both arches, closure of any residual spaces of missing teeth, and establishment of a functional molar relationship. After taking into consideration the age of the patient, the missing mandibular left molar and her occlusal status, a combined surgical-orthodontic approach was decided upon. Both lower deciduous molars were extracted under local anesthesia. Two metal bands were cemented to the maxillary first permanent molars, and a Quad-Helix appliance was inserted in the palatal tubes of the bands. Eight months later, all mandibular permanent premolars had fully erupted, and a new panoramic examination showed that the impacted molar was still at the same height (Figure 3).

Fixed orthodontic appliances (MBT, Dynaflex^®^, 10403 International Plaza Drive, St.Ann., MO, USA) were placed in the mandibular arch. After three months of active orthodontic treatment, initial alignment was achieved, and exposure of the impacted tooth was performed. A muccoperiostal flap was raised under local anesthesia, and a communication was established between the cystic cavity and the oral cavity. A specimen of the cyst was sent for biopsy. The crown of the impacted tooth was exposed, and a conventional molar attachment was bonded on the buccal side perpendicular to the long axis of the second molar with a self-etching adhesive. To upright the second molar, elastic traction was applied.

Histopathological examination confirmed the diagnosis of a dentigerous cyst.

**Figure 4:**
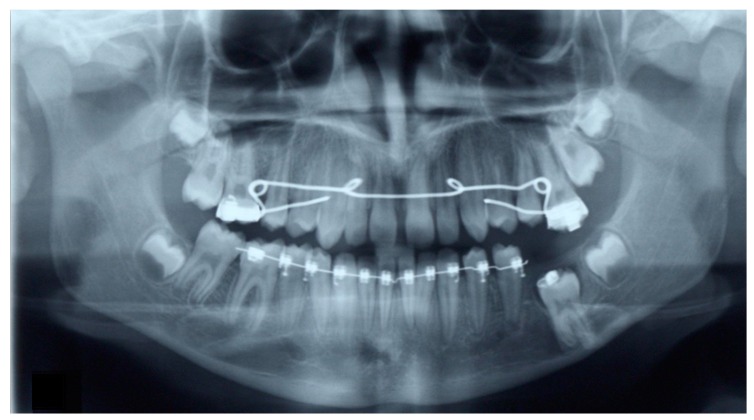
Panoramic radiograph two months after the surgical exposure of the first permanent mandibular molar. Note the reduction in size of the former cystic cavity and the more favorable axis of the impacted tooth.

**Figure 5:**
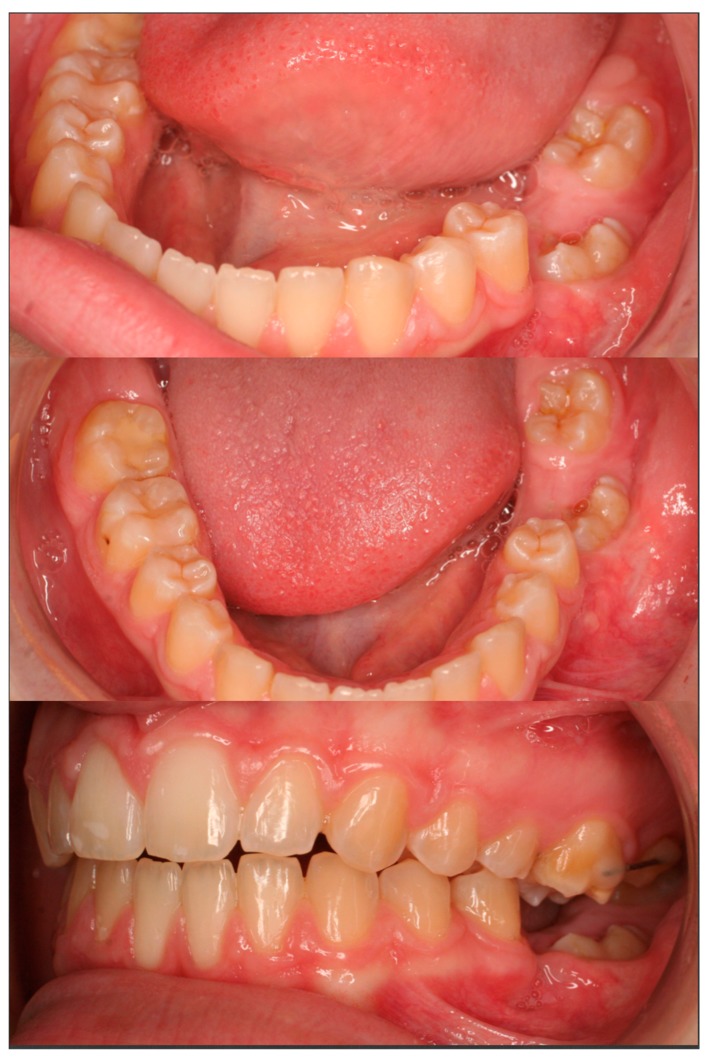
Immediately after debonding of the fixed orthodontic appliances of both dental arches. The mandibular left first molar was left to erupt spontaneously. Note that the maxillary molars are attached to one another via a stainless-steel wire in order to prevent further eruption of the first maxillary molar.

After two months of orthodontic extrusion, radiographic examination showed a significant reduction in the size of the cystic cavity and a more favorable position of the first molar (Figure 4). Orthodontic traction continued for 16 more months, while the upper dental arch was also treated with fixed orthodontic appliances. Eighteen months after the surgical procedure, the first molar had taken a very favorable position in the dental arch, thus debonding of the fixed appliances was decided due to the patient’s unwillingness to continue with the fixed appliance treatment. The previously impacted molar was left to erupt spontaneously, while the upper left molars were stabilized in order to prevent over-eruption (Figure 5).

**Figure 6:**
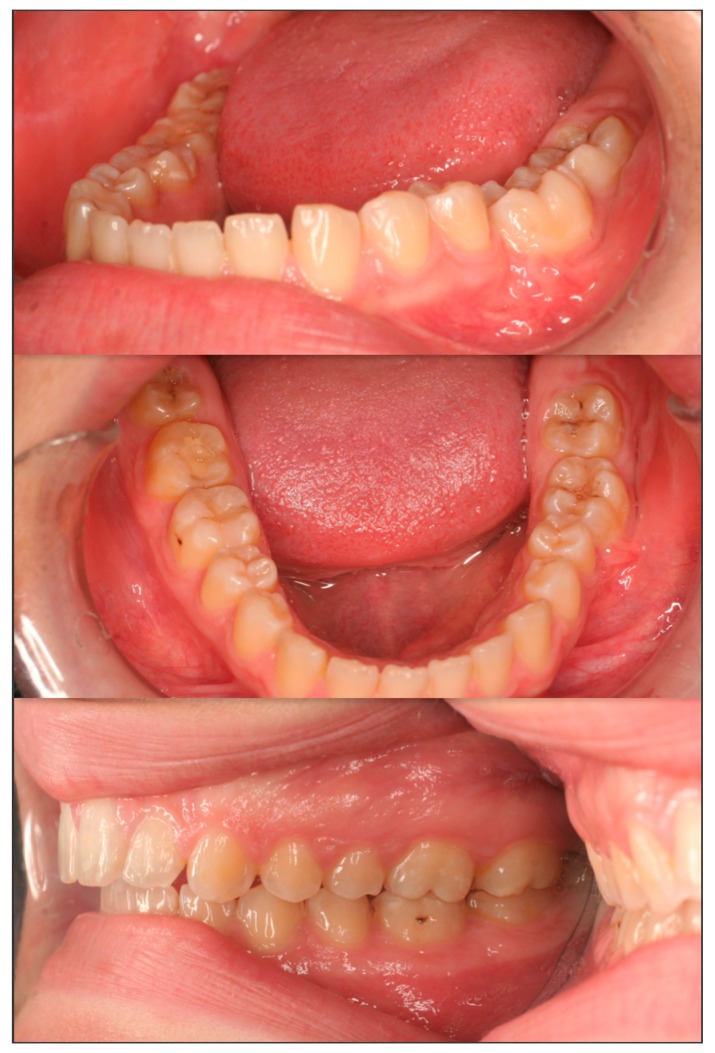
14 months after debonding of the fixed orthodontic appliances. The first mandibular left molar has fully erupted and is in occlusion with the maxillary first molar.

The patient reported for follow-up appointments every two months, and finally, 14 months after the debonding of the fixed appliances, the molar had fully erupted (Figure 6). A stable occlusion was achieved, both arches were well aligned, and there were no residual spaces.

**Figure 7:**
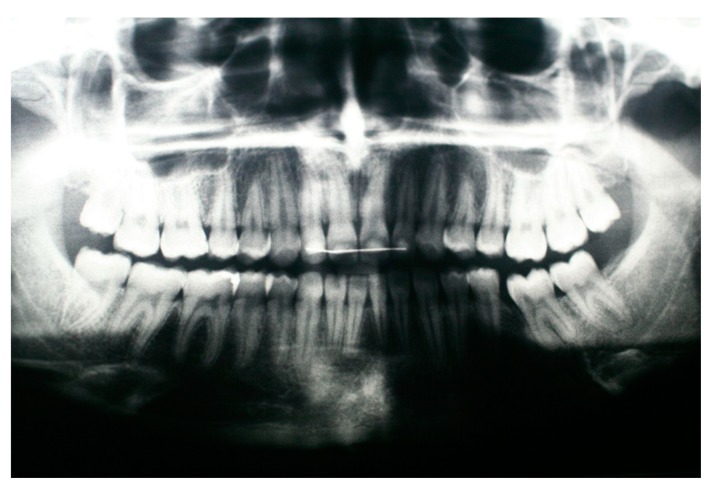
Panoramic radiograph 14 months after the debonding of the fixed orthodontic appliances and 32 months after the marsupialization of the dentigerous cyst.

Radiographic examination revealed an almost vertical position of the mandibular first molar and normal trabecular bone surrounding the previously impacted tooth (Figure 7).

## 3. Discussion

For the treatment of the dentigerous cyst, the possible alternatives were enucleation, marsupialization, or decompression of the cyst via fenestration. Marsupialization is a rather conservative treatment modality for the treatment of dentigerous cysts, as it decreases the risk of jaw fracture and nerve damage [18]. Moreover, it is the treatment of choice when the treatment plan involves preservation of the impacted tooth, in contrast with enucleation, where the involved tooth is extracted. Also, marsupialization, when compared to decompression of the cyst via fenestration, has the advantage of promoting the spontaneous tooth eruption of the tooth that is associated with the cyst [19]. However, in patients over 10 years old, spontaneous eruption does not often occur, thus orthodontic treatment is almost always necessary in order to guide the involved tooth to its final position in the dental arch in cases that the tooth is not extracted with the cyst [20]. Several orthodontic modalities have been described to guide the eruption of an impacted tooth, including segmental springs, diverse spring designs, elastic traction, or even mini implants [21,22,23]. Both segmental springs and diverse spring designs require complex mechanics and may cause tissue irritation to the patient. Instead, elastic traction can be quite acceptable from the patient and would be adequate in our case, as the long axis of the impacted tooth did not present severe angulation. Mini implants can be quite helpful in the uprighting process, but due to their cost and the surgical process involved, the use of this technique was rejected.

Impacted first molars associated with a dentigerous cyst are very rare, thus little information is available in the dental literature. Moreover, agenesis of the lower left second molar is also a very rare entity [24]. Usually, dentigerous cysts are associated with impacted teeth, mandibular third molars being the most commonly affected teeth, followed by the permanent maxillary canine and the permanent maxillary third molar [7]. Less commonly, it can occur in central incisors, yet the occurrence of such a cyst with a permanent first molar is still uncommon and comprises about 1.1% of all dentigerous cysts [25]. Male patients are more frequently affected, especially during the second and the third decade of life [6].

It is known that dentigerous cysts may present complications such as pathological fractures [26,27,28] or transformation, either malignant or ameloblastic. Even though malignant transformation is rare, such cysts should be either enucleated or marsupialized [29,30]. In our case, even though there was lack of any symptoms, the cyst size and the location made the decision for early treatment necessary. Histological examination was considered essential to confirm the clinical and the radiographical diagnosis.

The clinical and radiological criteria for selecting the appropriate treatment modality relate to the following: cyst size and location, dentition involved and stage of the root development of the involved tooth, the position of the involved tooth in the bone and relation to the adjacent teeth or anatomical structures and age of the patient [31]. Combination of enucleation and complete removal of the involved tooth is preferred in cases involving a single tooth impaction. For instance, this is usually the treatment of impacted third molars in adults as they have no function; however, when other teeth are impacted, removal of the tooth is not often in the patient’s best interests or desire, especially in children as removal of such teeth may have functional, esthetic as well as psychological consequences [15]. In our case, the cyst was marsupialized because the associated tooth was vital in order to achieve a functional occlusion and an esthetic result.

Combined orthodontic and surgical treatment was effective in preventing not only nerve damage but also periodontal breakdown of the impacted tooth and the adjacent premolar. The marsupialization allowed for some bone filling of the residual cavity as the cyst decompressed [15,28,32].

Different treatment modalities such as surgical repositioning and extraction of the impacted tooth induce a higher risk of complications. Surgical repositioning or transplantation may provoke root resorption, ankylosis, or even pulp necrosis and therefore should be the treatment of choice only in cases where orthodontic treatment is contraindicated [33,34].

Extraction of the impacted tooth to let the non-impacted molars erupt does not always have the optimized result, as the other molars may incline towards the extraction site [35]. In our case, extraction would have had destructive effects for the occlusion, as the second molar was missing.

Cooperation of the patient is fundamental when marsupialization is the treatment of choice, as oral hygiene and long-term patience is crucial for treatment success. The orthodontic appliances can irritate the adjacent mucosa and cause pain to the patient [36]. The fact that elastic traction was used for the uprighting of the molar until it reached the gingival margin made it easier and more convenient for the patient in order to achieve good oral hygiene.

## 4. Conclusions

We showed cyst marsupialization together with orthodontic molar uprighting to be effective in treating a dentigerous cyst associated with an impacted mandibular first molar. Such an interdisciplinary approach makes treatment easier, greatly reduces the risk of postsurgical complications, and appears to be advantageous in terms of the periodontal health of adjacent teeth. In addition, it is a conservative surgical approach that is generally preferred by the patients, as it preserves the function and the esthetic values and also prevents the adolescent from psycho-social trauma associated with tooth loss. However, such an approach does require close cooperation—not only among practitioners but also between the patient and the practitioners—in order to monitor the healing of the lesion, the maintenance of adequate oral hygiene, and the compliance of the patient with the long-term evaluation of the uprighting of the tooth in its final position.
